# Cowden syndrome-associated germline *SDHD* variants alter PTEN nuclear translocation through SRC-induced PTEN oxidation

**DOI:** 10.1093/hmg/ddu425

**Published:** 2014-08-22

**Authors:** Wanfeng Yu, Xin He, Ying Ni, Joanne Ngeow, Charis Eng

**Affiliations:** 1 Genomic Medicine Institute, Learner Research Institute; 2 Taussig Cancer Institute; 3 Stanley Shalom Zielony Institute of Nursing Excellence, Cleveland Clinic, Cleveland, OH 44195, USA; 4 Department of Genetics and Genome Sciences; 5 CASE Comprehensive Cancer Center; 6 Department of Epidemiology and Biostatistics, Case Western Reserve University, Cleveland, OH 44116, USA and; 7 Oncology Academic Clinical Program, Duke-NUS Graduate Medical School, Singapore 169610, Singapore

## Abstract

Germline mutations in the *PTEN* tumor-suppressor gene and germline variations in succinate dehydrogenase subunit D gene (*SDHD*-G12S, *SDHD*-H50R) are associated with a subset of Cowden syndrome and Cowden syndrome-like individuals (CS/CSL) and confer high risk of breast, thyroid and other cancers. However, very little is known about the underlying crosstalk between SDHD and PTEN in CS-associated thyroid cancer. Here, we show *SDHD*-G12S and *SDHD*-H50R lead to impaired PTEN function through alteration of its subcellular localization accompanied by resistance to apoptosis and induction of migration in both papillary and follicular thyroid carcinoma cell lines. Other studies have shown elevated proto-oncogene tyrosine kinase (SRC) activity in invasive thyroid cancer cells; so, we explore bosutinib, a specific inhibitor for SRC, to explore SRC as a mediator of SDH-PTEN crosstalk in this context. We show that SRC inhibition could rescue SDHD dysfunction-induced cellular phenotype and tumorigenesis only when wild-type PTEN is expressed, in thyroid cancer lines. Patient lymphoblast cells carrying either *SDHD*-G12S or *SDHD*-H50R also show increased nuclear PTEN and more oxidized PTEN after hydrogen peroxide treatment. Like in thyroid cells, bosutinib decreases oxidative PTEN in patient lymphoblast cells carrying *SDHD* variants, but not in patients carrying both *SDHD* variants and *PTEN* truncating mutations. In summary, our data suggest a novel mechanism whereby *SDHD* germline variants *SDHD*-G12S or *SDHD*-H50R induce thyroid tumorigenesis mediated by PTEN accumulation in the nucleus and may shed light on potential treatment with SRC inhibitors like bosutinib in *PTEN*-wild-type *SDHD*-variant/mutation positive CS/CSL patients and sporadic thyroid neoplasias.

## INTRODUCTION

Thyroid cancer is the most rapidly rising incident cancer in women and the second most rapidly rising incident cancer in men in the United States. In 2013, about 60 220 new cases of thyroid cancer were diagnosed, compared with 23 000 new cases in 2005 (http://www.cancer.org/cancer/thyroidcancer/detailedguide/thyroid-cancer-key-statistics). More than 1850 patients die from it, a number that is rising annually despite aggressive multi-modality therapy. It is critical and necessary to identify and characterize genes and signal transduction pathways that play a role in initiation, invasion and progression of thyroid cancer. Inherited factors represent an earliest etiologic step of thyroid cancer initiation. Cowden and Cowden-like syndrome (CS/CLS) represent a prototype of thyroid-associated heritable cancer syndromes.

Germline mutations in *PTEN* are associated with subsets of CS and Bannayan–Ruvalcaba–Riley syndrome, which predispose to breast and thyroid neoplasias (1–4). CS is an autosomal dominant disorder that is difficult-to-recognize, and is therefore, under-diagnosed (5). In addition to breast and thyroid carcinomas, recent work has shown elevated risks of endometrial, renal and colon cancers as well as melanomas in individuals with *PTEN* germline mutations (3). Accumulating studies suggest that subcellular localization of PTEN may play an important role in cell growth and tumorigenesis (6–8). PTEN nuclear accumulation in response to oxidative stress has been reported as one of the plausible mechanisms (9–11).

Mitochondrial complex II or succinate dehydrogenase (SDH) lies at the crossroad of electron transport and the Krebs cycle. Germline compound heterozygous *SDHx* mutations are associated with neuropathy, cardiopathy and hepatopathy and are lethal in infancy (12). Germline heterozygous *SDHx* mutations are associated with pheochromocytoma/paraganglioma (PC/PGL) syndromes (13–15). We found germline *SDHB/D* variants in ∼10% of *PTEN* mutation negative CS/CSL, associated with increased thyroid carcinoma prevalence compared with those with only germline *PTEN* mutations (16,17). Among the *SDHD* variants from CS patients, ∼95% are either *SDHD-*G12S or *SDHD*-H50R (17). We have shown that CS-related *SDHx* variants are associated with elevated reactive oxygen species (ROS), hyperactivated hypoxia-inducible factor (HIF), induced lipid peroxidation and disrupted mitochondrial metabolites in CS/CSL patient-derived lymphoblastoid cells (18). Therefore, the dysfunction from these *SDHx* variants could potentially affect PTEN signaling leading to such CS phenotypes as thyroid cancer.

Besides hypoxia-related stress, oncogenes such as exogenous murine proto-oncogene tyrosine kinase (Src) transfected in HEK293 cells have been reported to activate HIF (19). SRC plays critical roles in cell proliferation, survival, motility, migration, cell-matrix adhesion dynamics and regulation of the cytoskeleton via multiple downstream signaling pathways. SRC family kinases are overexpressed or hyperactivated in human neoplasms including breast, colorectal, prostate, pancreas, head and neck and lung, as well as thyroid carcinomas (20). Bosutinib, identified as a SRC kinase inhibitor, is effective in preventing de-differentiation, reducing tumor growth, invasion and distant metastasis in multiple xenograft tumor models (21–23).

In this study, therefore, we sought to address our hypothesis that CS/CSL-associated germline variants in *SDHD* could alter PTEN nuclear localization through SRC-induced PTEN oxidation in thyroid cancer cells.

## RESULTS

### *SDHD* variants in thyroid cancer cells lead to increased oxidized PTEN and PTEN accumulation in nuclei

**Figure 1.**
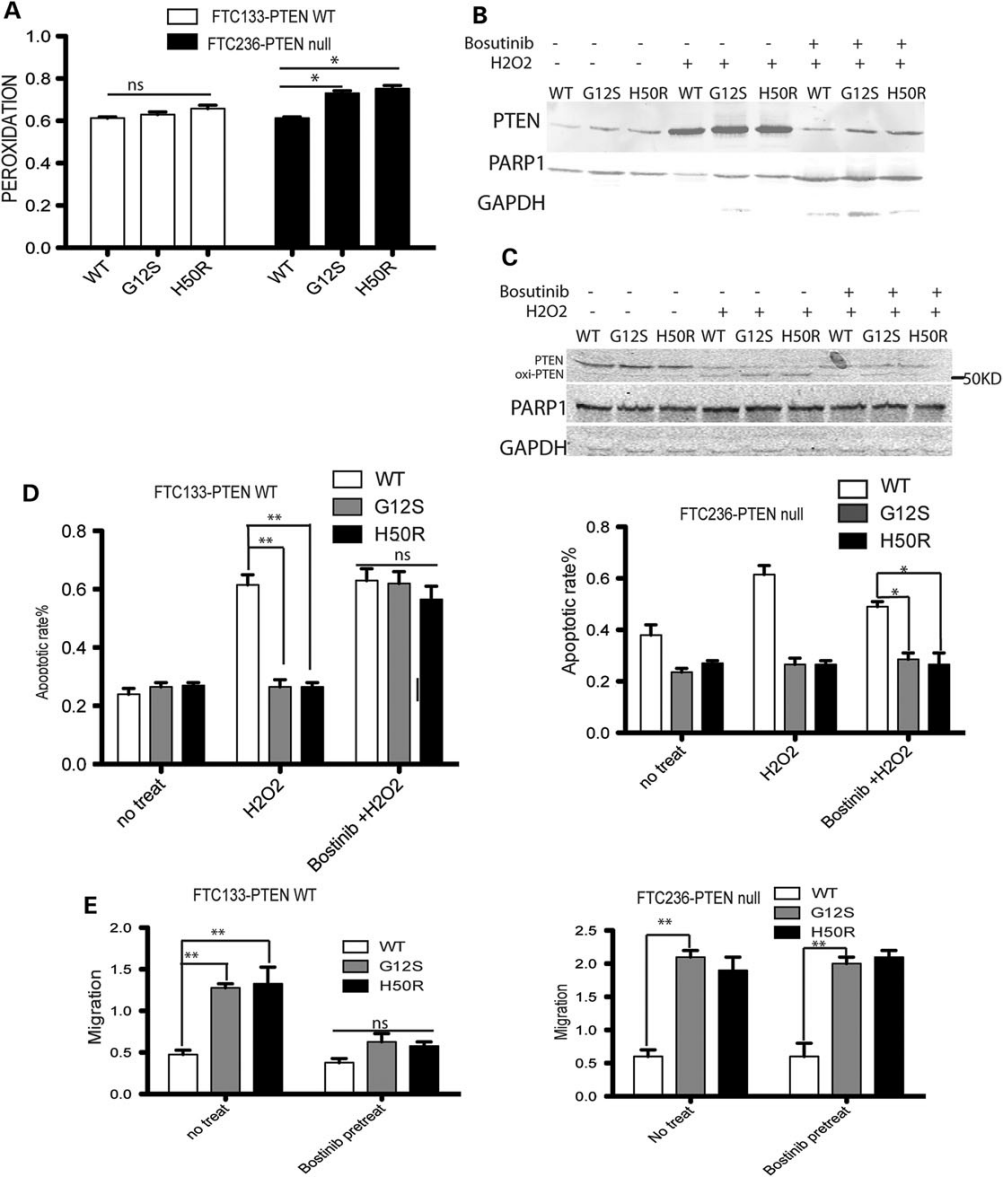
Accumulation of oxidized nuclear PTEN from SDHD-G12S and SDHD-H50R was abolished with bosutinib pretreatment in FTC133-PTEN wild-type cell, but not in FTC236-PTEN null cells. (**A**) Lipid peroxidation levels were measured in FTC133-PTEN wild-type and FTC23-PTEN null cells transfected with *SDHD*-wild type, -G12S and -H50R. Means ± SE from three representative experiments with *n* = 3 in each condition. (**B**) Nuclear PTEN was measured in FTC133-PTEN wild-type cells transfected with *SDHD*-wild type, -G12S and -H50R, respectively, after H_2_O_2_ exposure with or without bosutinib pretreatment. Western blots were performed three independent times, and the best blot was shown. (**C**) Nuclear oxidized PTEN was measured in FTC133-PTEN wild-type cells after transfection with wild type, G12S and H50R *SDHD* constructs after H_2_O_2_ exposure with or without bosutinib pretreatment. Western blots were performed four independent times and the best blot was shown. (**D**) Apoptotic rates in FTC133-PTEN wild-type cells transfected with *SDHD*-wild type, -G12S and -H50R with or without H_2_O_2_, with or without bosutinib pretreatment (left). Apoptotic rates in FTC236-PTEN null cells with *SDHD*-wild type, -G12S and -H50R with or without H_2_O_2_, with or without bosutinib pretreatment. Results are the mean ± SE of two independent experiments with *n* = 3 under each condition. (**E**) Wound healing migration rates were measured in FTC133-PTEN wild type containing SDHD-WT, -G12S or -H50R with/without bosutinib pretreatment (left) and in FTC236-PTEN null transfected with *SDHD*-wild type, -G12S and -H50R with or without bosutinib pretreatment (right). The results are the mean ± SE of three independent experiments with *n* = 5 in each condition. **P < 0.005, *P < 0.05.

We previously found that cellular ROS is significantly increased in CS/CSL patient samples harboring germline *SDHx* variants compared with normal controls (16). To determine if the *SDHD* variants in thyroid cancer cells can result in damage to lipids by, e.g. lipid peroxidation, we measured the byproducts of polyunsaturated fatty acid peroxides upon decomposition, namely, malondialdehyde (MDA) and 4-hydroxyalkenals (24) in two thyroid cancer cell lines follicular thyroid carcinoma (FTC) 133-PTEN wild-type and FTC236-PTEN null cells transfected with *SDHD*-G12S or -H50R. Compared with control SDHD-wild-type (25) transfected cells, no significant increase of lipid peroxidation was observed in FTC133-PTEN wild-type cells with *SDHD*-G12S or *SDHD*-H50R. In contrast, a slight increase in lipid peroxidation was observed in *SDHD*-G12S or *SDHD*-H50R transfected FTC236-PTEN null cells (Fig. 1A). We therefore surmised that wild-type PTEN in FTC133-PTEN wild type could play a protective role against SDHD variant-induced oxidative stress.

Western blots of the nuclear fraction of PTEN showed dramatic increases of nuclear PTEN in both control and *SDHD*-G12S or *SDHD*-H50R-transfected FTC133-PTEN wild-type cells after ROS exposure, which could be abolished by pretreatment with the SRC kinase inhibitor bosutinib (Fig. 1B). To explore the oxidative status of the accumulated nuclear PTEN under oxidative stress, fractionated nuclear proteins were harvested with *N*-ethylmaleimide (26), which maintains disulfide bonds in their oxidized form. Oxidation of PTEN protein is known to induce the formation of a disulfide bound between Cys71 and Cys124. After exposure to H_2_O_2_, the *SDHD*-G12S or *SDHD*-H50R-transfected FTC133-PTEN wild-type cells showed more oxidized nuclear PTEN compared with *SDHD*-wild-type-transfected cells (Fig. 1C). The oxidized nuclear PTEN from H_2_O_2_ exposure was dramatically decreased in bosutinib-pretreated cells (Fig. 1C). These observations suggest that ROS-induced PTEN nuclear accumulation and increased oxidized nuclear PTEN are mediated by SRC activation.

### Reduced apoptosis and induced migration in *SDHD*-G12S or *SDHD*-H50R-transfected thyroid cancer cells are rescued by pretreatment with SRC kinase inhibitor only in FTC133-PTEN wild-type cell line

Expression of *SDHD*-G12S or -H50R in both FTC133-PTEN wild-type and FTC236-PTEN null cell lines led to resistance to apoptosis upon H_2_O_2_ exposure compared with dramatic increases in apoptosis in *SDHD*-wild-type-transfected FTC cells. Pretreatment with bosutinib in FTC133-PTEN wild-type transfected with either *SDHD*-G12S or -H50R showed similar levels of apoptosis upon H_2_O_2_ exposure as FTC133-PTEN wild-type cells with transfected *SDHD*-WT construct (Fig. 1D). However, bosutinib pretreatment in FTC236-PTEN null cells containing either of the *SDHD* variants (-G12S, -H50R) did not change their resistance to apoptosis upon H_2_O_2_ exposure (Fig. 1D). Similarly with *SDHD* variant-harboring cells, induced migration was observed in FTC133-PTEN wild-type cells transfected with either *SDHD*-G12S or *SDHD*-H50R, which was effectively blocked by bosutinib pretreatment. In FTC236-PTEN null cell lines, bosutinib did not affect the difference observed in the migration rates between *SDHD*-G12S/H50R and *SDHD*-WT cells (Fig. 1E). These data suggest that SRC inhibition could block *SDHD*-G12S/H50R-mediated resistance to apoptosis and increased migration specifically through PTEN expression.

### SRC inhibition abolished resistance to apoptosis and increased migration in *SDHD*-silenced FTC133-PTEN wild-type cells, but not FTC236-PTEN null cells

**Figure 2.**
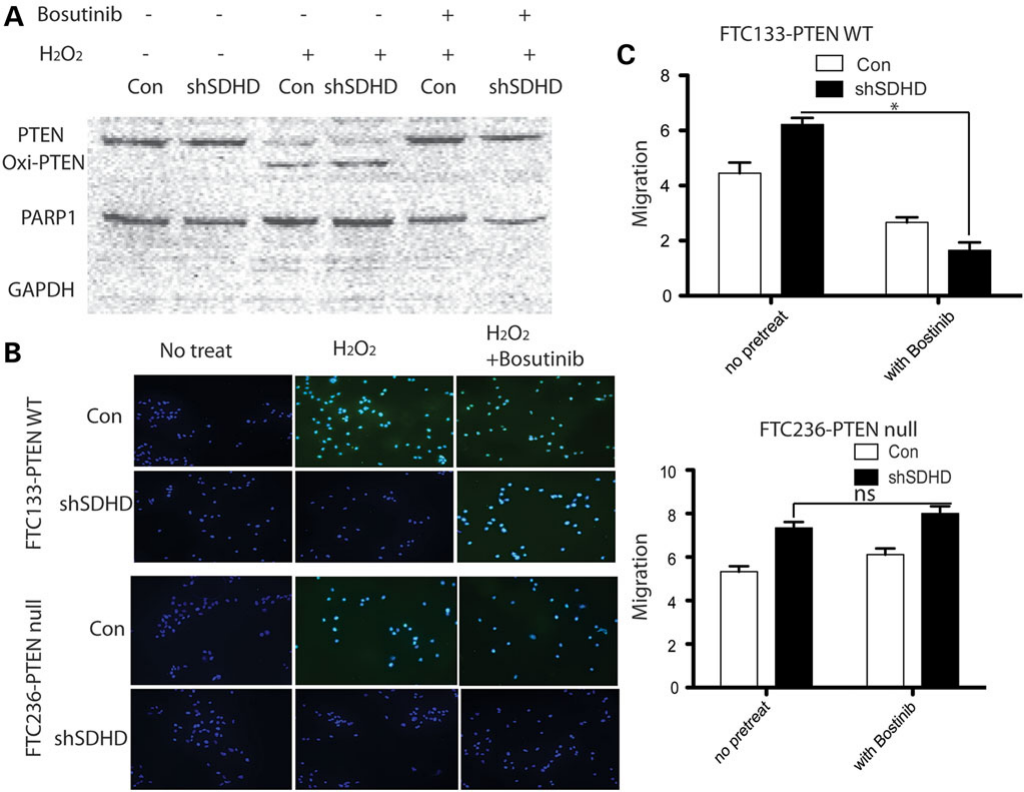
Bosutinib pretreatment abolished accumulation of nuclear oxidized PTEN, rescuing tumorigenic phenotypes in shSDHD-transfected FTC133-PTEN wild-type cells, but not in shSDHD-transfected FTC236-PTEN null cells. (**A**) Nuclear oxidized PTEN was measured by western blot in FTC-133-PTEN wild-type cells, in the presence or absence of H_2_O_2_, with or without bosutinib pretreatment. Western blots were performed independently five times. (**B**) Wound healing migration rates were measured in shSDHD-transfected FTC133-PTEN wild-type cells and shCON-transfected FTC133-PTEN wild-type cells with or without bosutinib pretreatment (upper); wound healing migration rates were measured in shSDHD-transfected FTC236-PTEN null and shCON-tranfected FTC236-PTEN null cells with or without bosutinib pretreatment (lower). The results are the mean ± SE of three independent experiments with *n* = 5 in each condition. *P < 0.05. (**C**) TUNEL staining showing apoptotic cells in FTC133-PTEN wild-type cells (upper panels), and in FTC236-PTEN null cells (lower panels) transfected with either shSDHD or shCON, in the presence or absence of H_2_O_2_, with or without bosutinib. Magnification: ×200. Experiments were performed four independent times.

To confirm our observations in *SDHD*-variant-bearing FTC133-PTEN wild-type and FTC236-PTEN null cell lines, we silenced SDHD expression in FTC133-PTEN wild-type and FTC236-PTEN null cell lines. More oxidized PTEN was observed in the nuclear fraction upon H_2_O_2_ treatment in FTC133-PTEN wild-type cells. Bosutinib pretreatment was associated with lack of oxidized PTEN in both *SDHD*-silenced and control (sham transfected) cells (Fig. 2A). Consistent with our observations in *SDHD*-G12S/H50R cells, apoptosis induced by H_2_O_2_ was noted in *SDHD*-silenced FTC133-PTEN wild-type cells that were bosutinib pretreated. Apoptosis resistance in *SDHD*-silenced FTC236-PTEN null cells was not affected by bosutinib (Fig. 2B). Induced migration in *SDHD*-silenced FTC236-PTEN null cells was dramatically decreased with bosutinib pretreatment, whereas *SDHD*-silenced FTC236-PTEN null cells showed no significant change in migration even with bosutinib pretreatment (Fig. 2C).

### SRC inhibition associated with apoptosis and reduced migration of FTC133-PTEN wild-type cells but not FTC236-PTEN null cells

**Figure 3.**
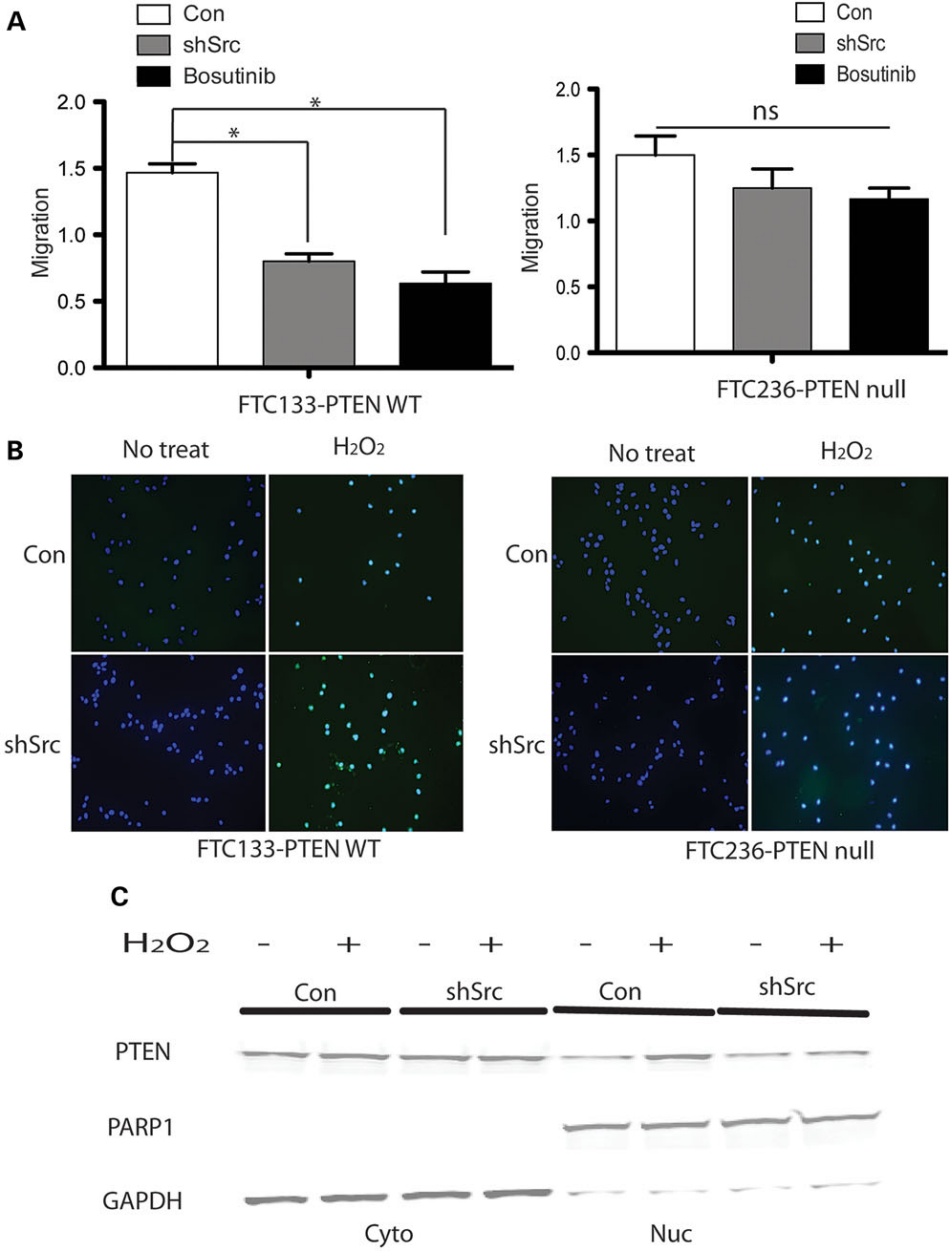
Short-hairpin SRC reduced migration and induced apoptosis in FTC133-PTEN wild-type cells, but not in FTC236-PTEN null) cells. (**A**) Wound healing migration rates normalization in SRC-silenced FTC133-PTEN wild-type cells (left) and FTC236-PTEN null) cells (right). The results are the mean ± SE of three independent experiments with *n* = 5 in each condition. *P < 0.05. (**B**) TUNEL staining of apoptotic cells in SRC-silenced FTC133-PTEN wild-type cells with or without H_2_O_2_ exposure (left) and SRC-silenced FTC236-PTEN null cells with or without H_2_O_2_ exposure (right). Magnification: ×200. Experiments were performed five independent times. (**C**) Nuclear PTEN in SRC-silenced FTC133-PTEN wild-type cells and control FTC133-PTEN wild-type cells (transfected with empty vector) with or without H_2_O_2_, with or without bosutinib. Western blots were performed four independent times.

SRC-silenced FTC133-PTEN wild-type cells showed decreased migration compared with shCon FTC133-PTEN wild-type cells. No significant difference was observed between SRC-silenced FTC236-PTEN null cells and control FTC236-PTEN null cells (transfected with empty vector) (Fig. 3A). Upon H_2_O_2_ treatment, more induced apoptosis was observed in SRC-silenced FTC133-PTEN wild-type compared with FTC133-PTEN wild-type control cells (with empty vector). No significant difference in apoptosis was observed between SRC-silenced FTC236-PTEN null and FTC236-PTEN null control cells (with empty vector) (Fig. 3B).

Nuclear and cytoplasmic fractions from FTC133-PTEN wild-type cells showed increased nuclear PTEN in control cells upon H_2_O_2_ treatment, whereas H_2_O_2_ did not induce nuclear accumulation of PTEN in SRC-silenced FTC133-PTEN wild-type cells (Fig. 3C). These observations suggest that SRC-induced apoptosis and reduced migration are mediated by PTEN, at least in thyroid cancer cells.

### SRC inhibition blocks H_2_O_2_-induced HIF1α activation in thyroid cancer cells

**Figure 4.**
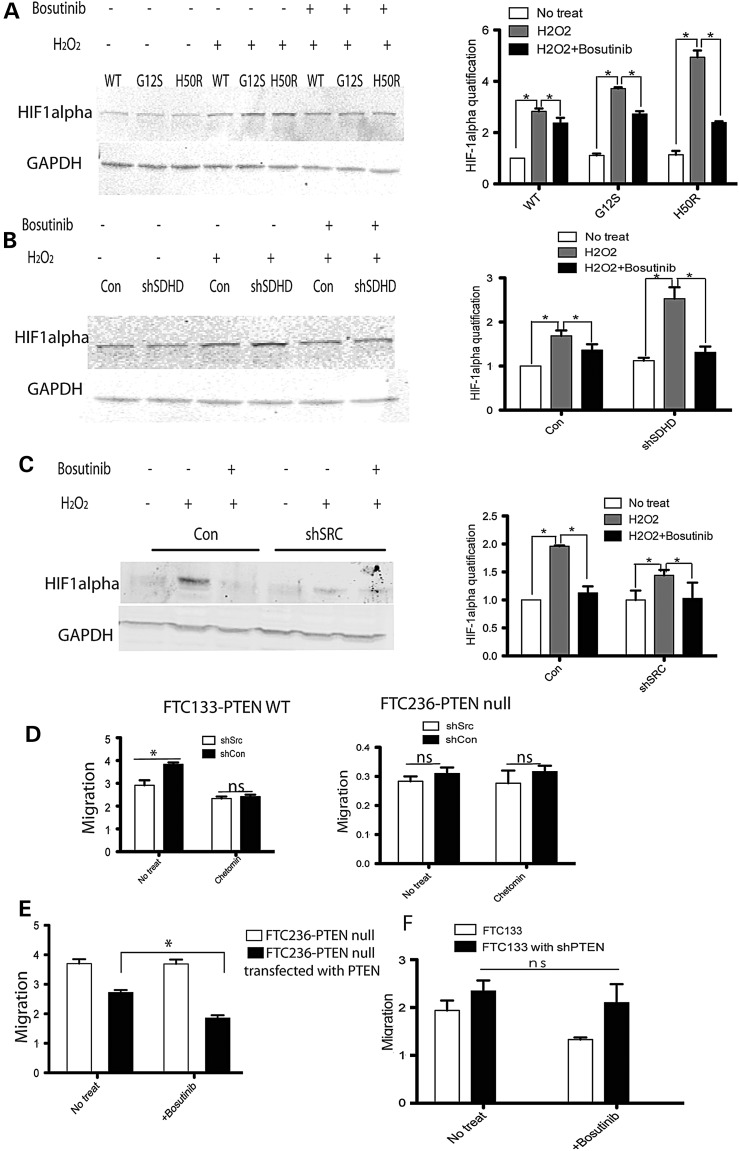
HIF-1α was stabilized with H_2_O_2_ treatment and inhibited by SRC inhibitor bosutinib in FTC133-PTEN wild-type cells. (**A**) Western blot of HIF-1α expression in SDHD-wild type, -G12S or -H50R transfected into FTC133-PTEN wild-type cells, with or without H_2_O_2_, with or without bosutinib. The bar graph summarizes the relative expression of three independent experiments. (**B**) HIF-1α expression in FTC133-PTEN wild-type cells after shSDHD transfection or shCON control-transfection with or without H_2_O_2_, with or without bosutinib. The bar graph summarizes the relative expression of three independent experiments. (**C**) HIF-1α expression in FTC133-PTEN wild-type cells transfected with shSRC or shCON control construct, with or without H_2_O_2_, with or without bosutinib. The bar graph summarizes the relative expression of two independent experiments. (**D**) Wound healing migration rates with or without HIF-1 inhibitor chetomin pretreatment in FTC133-PTEN wild-type cells (left) and in FTC236-PTEN null cells (right). The results are the mean ± SE of two independent experiments with *n* = 4 in each condition. (**E**) Wound healing migration rates in wild-type PTEN transfected FTC236-PTEN null cells with or without bosutinib. The results are the mean ± SE of three independent experiments with *n* = 3 in each condition. (**F**) Wound healing migration rates in PTEN knockout FTC133-PTEN wild-type cells with or without bosutinib. The results are the mean ± SE of two repeated experiments with *n* = 3 in each condition. *P < 0.05.

To further identify the effect of our SRC kinase inhibitor on thyroid cancer cell lines, we examined the activation of HIF-1α by ROS with or without SRC inhibition. In FTC133-PTEN wild-type cells expressing SDHD-G12S or SDHD-H50R, H_2_O_2_ treatment dramatically induced HIF-1α expression, with the levels of increased expression in SDHD-G12S or SDHD-H50R-containing cells much more dramatic compared with those from control (SDHD-WT) cells. Pretreatment with bosutinib was associated with blockade of the increased HIF-1α expression from H_2_O_2_ exposure in both SDHD-G12S/H50R expressing cells and control (SDHD-wild-type) cells (Fig. 4A). Consistently, we observed further increases of HIF-1α expression in *SDHD*-silenced cells compared with control cells (transfected with empty plasmid), and this increased HIF-1α expression was also blocked by bosutinib pretreatment (Fig. 4B).

We subsequently examined changes of HIF-1α expression in SRC-silenced cells. Compared with non-SRC-silenced control cells, oxidative stress induced much less HIF-1α in SRC-silenced cells (Fig. 4C). These data corroborate our above observation that SDHD dysfunction-associated oxidative stress is mediated by SRC activation. To validate that SRC-associated HIF-1α activation leads to increased migration through PTEN, we used HIF-1α-specific inhibitor chetomin to pretreat SRC-silenced cells. Wound healing assay showed decreased migration in FTC133-PTEN wild type after HIF-1α inhibitor pretreatment, whereas chetomin did not affect migration rates in the native PTEN null FTC236 cells (Fig. 4D). To further validate PTEN expression mediated bosutinib-rescued tumorigenic phenotype in thyroid cancer cells, we transfected wild-type PTEN in FTC236-PTEN null cells and parallelly knockdown PTEN in FTC133-PTEN wild-type cells, then pretreated them with or without bosutinib before assaying their migration rate. As expected, we observed that pretreatment of bosutinib decreased migration rate in FTC236-PTEN null cells transfected with PTEN (Fig. 4E); no significant changes of migration observed from bosutinib pretreatment in PTEN knockdown FTC133 cells (Fig. 4F).

### SRC inhibition blocked nuclear PTEN accumulation and decreased migration in papillary thyroid cancer cells expressing *SDHDv*

**Figure 5.**
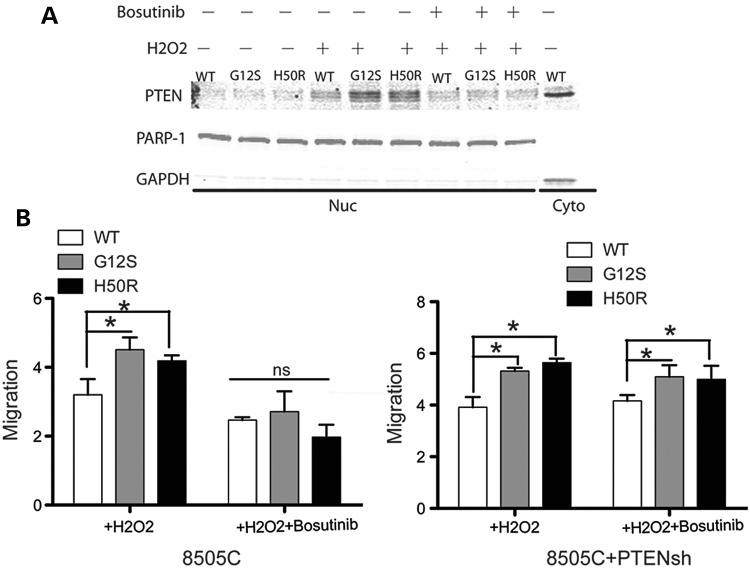
Accumulation of oxidized nuclear PTEN from SDHD-G12S and SDHD-H50R was abolished with bosutinib pretreatment in 8505C cells. (**A**) Nuclear PTEN was measured in 8505C cells transfected with *SDHD*-wild type, -G12S and -H50R, respectively, after H_2_O_2_ exposure with or without bosutinib pretreatment. (**B**) Wound healing migration rates normalization in papillary thyroid carcinoma (PTC) cell line 8505C cells transfected with *SDHD*-WT, -G12S, or -H50R, respectively, with/without Bosutinib treatment (left) and 8505C cells co-transfected with shPTEN and *SDHD*-WT, -G12S or H50R, respectively, with/without Bosutinib treatment (right). The results are the mean ± SE of four independent experiments with *n* = 5 in each condition. *P < 0.05, ns: not significant (*P* > 0.05).

To further determine if *SDHDv* affects PTEN function in papillary thyroid cancer cell line, we next transfected 8505C cells with SDHD wild-type, *SDHD*-G12S and *SDHD*-H50R plasmids, respectively. More nuclear PTEN was detected in 8505C cells transfected with *SDHD*-G12S and *SDHD*-H50R with H_2_O_2_ treatment. Consistent with FTC cell results, bosutinib pretreatment dramatically decreased nuclear PTEN accumulation (Fig. 5A). Wound healing migration assay showed increased migration in 8505C cells transfected with *SDHD*-G12S or *SDHD*-H50R, which were inhibited with SRC inhibition (Fig. 5B, left). When we knock down PTEN with shPTEN, SRC inhibitor bosutinib had no effect on migration in 8505C cells (Fig. 5B, right).

### Lymphoblastoid cell lines from SDHD variant positive CS patients showed elevated SRC activity and increased nuclear PTEN accumulation

**Figure 6.**
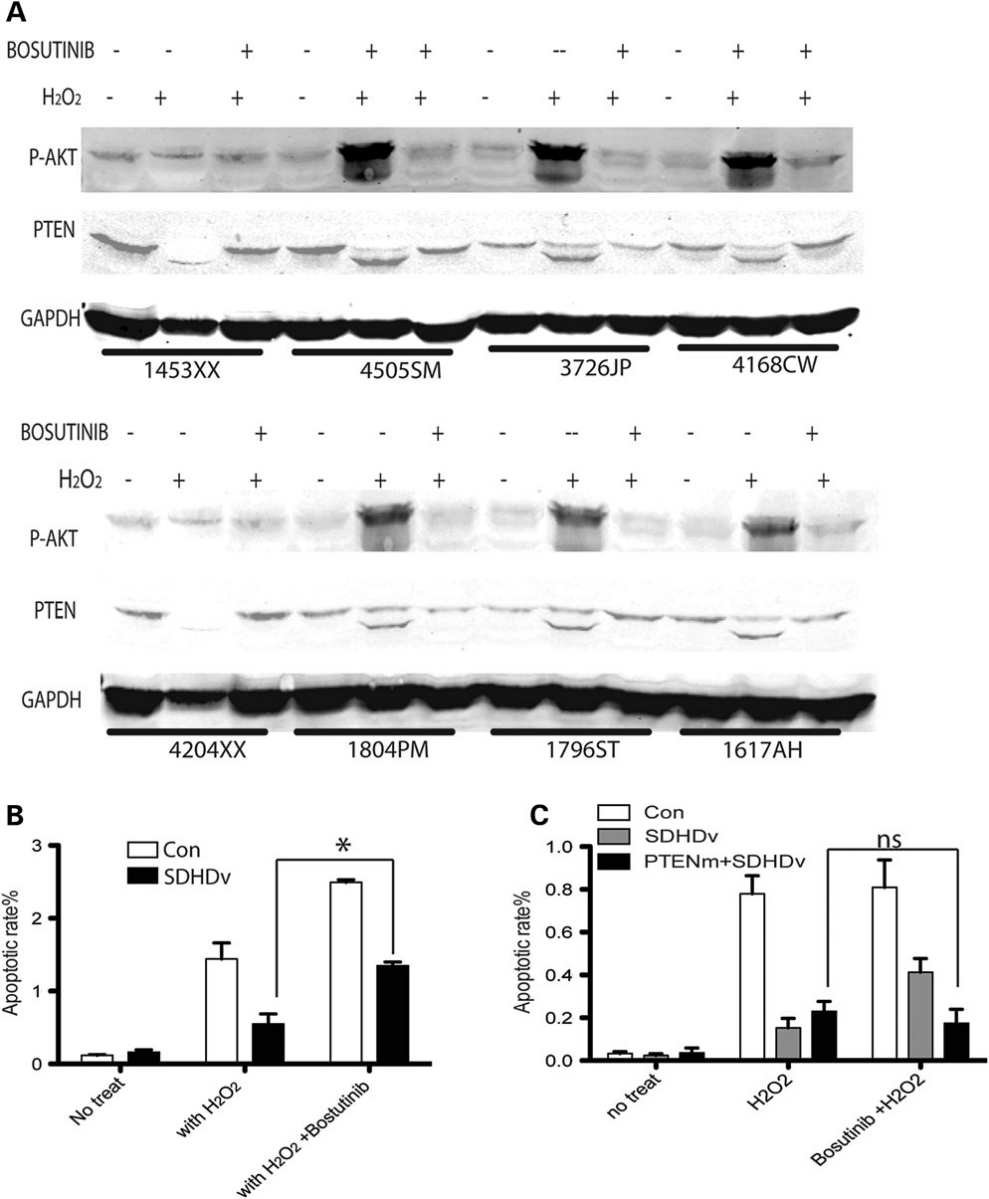
Bosutinib inhibits PTEN oxidation and induces apoptosis in CS patient LCL cells harboring either SDHD-G12S or H50R naturally occurring germline variants, but not in LCL cells with both *SDHD* variants and *PTEN* mutations. (**A**) Oxidized PTEN in LCLs with *SDHD*-G12S or *SDHD*-H50R variants. 1453XX and 4204XX are control LCLs; 4550SM, 3726JP, 1804PM and 1796ST are LCLs harboring germline *SDHD*-H50R; 4168CW and 1617AH are LCLs harboring germline *SDHD*-G12S. Experiments were performed three independent times. (**B**) Apoptotic rates in LCLs harboring either *SDHD*-G12S or H50R germline variants when exposed or not exposed to H_2_O_2_, with or without bosutinib (*n* = 6). The results are the mean ± SE of three independent experiments. (**C**) Apoptotic rates in LCLs harboring both *SDHD* variants and *PTEN* mutations (*n* = 3). The results are the mean ± SE of three independent experiments. *P < 0.05, ns: not significant (*P* > 0.05).

We then examined activation of SRC in LCL cells derived from a series of CS/CSL patients carrying either *SDHD*-G12S or *SDHD*-H50R variants. Compared with three LCLs derived from controls without *SDHD* germline variants, phosphorylation of tyrosine 418 on SRC, representing activated SRC, was dramatically higher in the *SDHD* variant-positive CS/CSL patients (Supplementary Material, Fig. S2A). PTEN western blot of the LCL nuclear and cytoplasmic fractionated proteins showed more nuclear PTEN in *SDHD*-variant carriers compared with those of the controls (Supplementary Material, Fig. S2B). Next, we pretreated the LCL cells with bosutinib before exposing them to H_2_O_2_. H_2_O_2_-induced oxidized PTEN was abolished by bosutinib pretreatment of the LCL cells derived from the CS patients (Fig. 6A). Fluorescence-activated cell sorting (FACS) analysis after propidium iodine staining showed that although H_2_O_2_ treatment could induce apoptosis in both healthy control LCL cells and patient LCL cells, the induced level of apoptosis in LCL from *SDHD* variant-positive patients was much lower than in control LCL cells. With bosutinib pretreatment, LCLs from CS patients have a greater increase of apoptosis rate with H_2_O_2_ exposure compared with cells without bosutinib treatment (Fig. 6B). Finally, we compared the effect of bosutinib on apoptotic rates in LCLs from CS patients with only SDHD variants and LCLs from CS patients with both *SDHD* variants and *PTEN* mutations. Bosutinib induced apoptosis in LCLs with *SDHD* variants (G12S or H50R). However, in three LCLs with both *SDHD* variants (either G12S or H50R) and *PTEN* mutations (insertion, truncation or early translation termination), which dramatically abolished PTEN function, no significant induction of apoptosis was observed (Fig. 6C). These data utilizing patient-derived cells further confirmed our *in vitro* cell line observation that SRC kinase inhibitor rescues tumorigenic phenotype required wild-type PTEN expression.

## DISCUSSION

We previously observed increased ROS and lipid peroxidation in CS/CSL patient-derived LCLs with germline *SDHx* variants. Here, we show that two *SDHD* variants *SDHD-G12S* and *SDHD-H50R* as well as *SDHD* knockdown lead to more oxidized PTEN and increased nuclear PTEN accumulation under peroxide-induced oxidative stress, which consequently induced resistance to apoptosis and increased migration in thyroid cancer cells. A SRC kinase inhibitor rescued the above tumorigenic phenotype only in PTEN-expressing thyroid cancer cells, but not in PTEN null cells.

The SDH enzyme, also known as mitochondrial complex II, is a highly conserved heterotetrameric protein complex comprising SDHA, SDHB, SDHC and SDHD. They are anchored to the inner membrane by SDHC and SDHD, which also provide the location for ubiquinone to bind and for electron transfer (27). The signal peptide of SDHD is composed of 56 amino acids, which provides the signal for SDHD to be located correctly in the inner membranes of mitochondria. Both variants G12S and H50R are located in the signal peptide of the SDHD preprotein. As SDHD is one of two important subunits anchoring the SDH complex in the membrane, these two variants (G12S and H50R) may lead to a mislocalization of the complex within the mitochondrial membrane or to altering the cleavage site of signal peptide, and subsequently dysfunction of the complex. We also observed similar cellular phenotypes in *SDHD* knockdown as in the *SDHD* variant-bearing (G12S, H50R) cells. This confirms our hypothesis that the two variants (G12S and H50R) located on SDHD signal peptide lead to complex II dysfunction and result in increased intracellular oxidative stress. A previous biological analysis in the tumor tissue from a PGL patient harboring a loss-of-function *SDHD* germline mutation (R22X) showed that SDH activity was abolished due to the mutation and associated with increased expression of HIF-1α (26). In addition, dysfunction of SDH in the tricarboxylic acid cycle inhibits prolyl-hydroxylation of HIF-1α and HIF-2α, which is an important step for their degradation (28). Our current observations consistently show that SDHD-G12S and SDHD-H50R, as well as SDHD knockdown, lead to functional insufficiency of SDH and increased stability of HIF-1α in thyroid cancer cell lines.

We observed that baseline levels of nuclear PTEN are higher in SDHD-G12S and SDHD-H50R FTC133-PTEN wild-type cells than in SDHD-wild-type FTC133-PTEN wild-type cells (Fig. 1B). This suggests that the G12S and H50R in SDHD alone could affect PTEN subcellular localization. With H_2_O_2_ exposure, this oxidative stress leads to more elevated levels of nuclear oxidized PTEN in SDHD-G12S and SDHD-H50R cells compared with that in SDHD-wild-type cells. Although mounting evidence shows increased oxidative stress and over-production of ROS are associated with cancer development, the exact mechanism is not very clear yet. We thus surmised that alteration in PTEN subcellular localization and oxidation status might be the crux of SDHD dysfunction, which provides a potential mechanism, and that is, oxidative stress affects PTEN subcellular localization and oxidation, resulting in PTEN-related apoptosis resistance and migration. Indeed, we showed that more oxidized PTEN accumulates in the nuclei of SDHD-G12S and -H50R transfected thyroid cancer cells upon oxidative stress. This is in agreement with the observation that oxidative stress stimulated Pten nuclear accumulation in mouse embryonic fibroblast cells (11). Oxidation of PTEN protein induces the formation of a disulfide bond between Cys71 and Cys124. Cys124 resides within the catalytic site of PTEN protein and its oxidation renders the protein enzymatically inactive. Increasing evidence from our laboratory has shown the crosstalk between the PTEN and mitochondrial signaling pathways. Impairment of PTEN function from PTEN oxidation has been reported previously (29–32), but the precise mechanism how variants and mutations in mitochondrial tumor suppressors such as *SDH* lead to carcinogenesis phenotypes in patients is not clear yet. Our current data show, for the first time, the link between SDH dysfunction and PTEN signaling in thyroid cancer development.

Src activity has been observed to be elevated in a murine model of thyroid cancer, and hyper-activation of Src is associated with HIF activity, at least in this model (20). In addition, aberrant somatic activation of SRC is frequent in human cancers including thyroid cancers. Over-activation of SRC increases cell growth and survival, as well as promotes cell migration, and decreases cell-matrix adhesion. Undoubtedly, inhibition of SRC is a promising target for controlling primary tumor growth, invasion and metastasis. Interestingly, we found that bosutinib, a specific SRC kinase inhibitor, can rescue tumorigenic phenotypes in thyroid cancer cells with PTEN expression, but not in PTEN null thyroid cancer cells. Our observations suggest that SDHD-variant-associated SRC activation leads to tumorigenic phenotypes in a PTEN-dependent manner. This PTEN inactivation associated signaling pathway might be the dominant signaling pathway induced by SDHD dysfunction in FTC133-PTEN wild-type cells since inhibition of SRC by either SRC knockdown or bosutinib was necessary and sufficient to rescue nuclear oxidized PTEN accumulation, inhibit migration and induce apoptosis. In PTEN null cells, neither SRC knockdown nor Bosutinib significantly altered the tumorigenic phenotypes upon oxidative stress. Thus, in FTC236-PTEN null cells, SDHD dysfunction-induced migration and reduced apoptosis must be mediated via signaling pathways other than SRC/PTEN signaling, which needs further investigation. Our observations here suggest that SRC inhibition could be potentially considered for tailoring treatment, at least for thyroid cancers, in the subset of CS/CSL patients carrying germline *SDH* variants in the context of wild-type PTEN.

Our observations here may offer a valuable clue exploring potential reasons of drug resistance to SRC inhibitors in some thyroid cancer patients. Ferri *et al.* (33) recently showed that the increased ratio of *LYN/PTEN* expression in leukocyte cells derived from leukemia patients was associated with resistance to tyrosine kinase inhibitor drugs. LYN is a tyrosine kinase belonging to the SRC family of kinases. They concluded that patients with a low level of *PTEN* mRNA expression or an increased expression of *LYN* showed lack of response to two tyrosine kinase inhibitor drugs imatinib and nilotinib. Thus, high expression of SRC family kinase together with low PTEN expression was suggested to be associated with resistance to tyrosine kinase inhibitor drugs. While they also reported that patients resistant to a SRC inhibitor dasatinib did not show any difference in the mRNA ratio of *LYN/PTEN* compared with healthy group controls. We considered these contradictory observations could be explained by tissue-specific differences or to the different SRC kinases being examined.

In summary, we have demonstrated that SDHD dysfunction does not change the absolute amount of PTEN expression, but instead is associated with increased oxidized PTEN, favoring the latter's nuclear location through activation of SRC, which weakens the PTEN anti-tumor function, thus resulting in apoptosis resistance and increased migration in both FTC and papillary thyroid carcinoma (PTC) cell lines. Therefore, we reveal a novel mechanism for SDH-associated thyroid cancer pathogenesis through SDH-mediated PTEN functional alteration. In addition, bosutinib, a SRC-specific inhibitor, abolished *SDHD*-variant-associated apoptosis resistance and migration acceleration in LCL derived from CS/CSL patients. Thus, our data also suggest potential uses of SRC inhibitors in *SDHD*-variant positive, *PTEN-wild-type* CS/CSL patients. Our observations here might also suggest that SDH dysfunction in the presence of PTEN alterations are a potential mechanism for drug resistance to SRC inhibitors in a broad range of sporadic malignancies. It would be interesting to analyze the predictive value of SDH and PTEN status in prospective clinical trials using SRC inhibitors and related molecules.

## MATERIALS AND METHODS

### Cell culture

Human FTC cell lines FTC133 (#94060901, purchased in 2011 from Sigma-Aldrich, Saint Louis, MO) with wild-type PTEN expression (FTC133-PTEN wild type) and FTC236 (FTC236-PTEN null) (#6030202, purchased in 2011 from Sigma-Aldrich, Saint Louis, MO) cells were grown in 50% Ham's F12, 50% DMEM supplemented with 10% fetal bovine serum, 1% glutamine, 0.25% insulin, 0.01U/mL TSH at 37°C with 5% CO_2_. The 8505C (#ACC219, purchased in 2011 from DSMZ, Braunschweig, Germany) human papillary thyroid cancer cell lines was grown in EMEM plus 1% non-essential amino acids and 10% fetal bovine serum at 37°C with 5% CO_2_. Immortalized lymphoblastoid cell lines (LCLs) obtained from CS patients or normal controls were grown in RPMI1640 supplemented with 20% fetal bovine serum and incubated at 37°C with 5% CO_2_.

### Plasmid transfection and stable cell line construction

Flag tagged SDHD-G12S, SDHD-H50R, SDHD wild type (25), shSDHD (Santa Cruz Tech., Santa Cruz, CA), shCon (plasmid with scrambled short-hairpin RNA), shSRC (Santa Cruz Tech., Santa Cruz, CA) and shPTEN (Sigma-Aldrich, Saint Louis, MO) plasmids were transfected in FTC133-PTEN wild-type, FTC236-PTEN null, 8505C cells, respectively, with Lipofectamine™ 2000 transfection reagent (Invitrogen, Carlsbad, CA). Fresh media was replaced 8 h post transfection. *SDHD*-G12S, *SDHD*-H50R and *SDHD*-WT transfected cells were selected by G418; shSDHD and shCon cells were selected with puromycin for 3 weeks and then single colonies were picked out and identified with either western blot or reverse transcriptase-PCR (Supplementary Material, Fig. S1).

### Lipid peroxidation measurement

The measurement of lipid peroxidation was performed using Lipid Peroxidation Microplate Assay Kit (Oxford Biomedical Research, Oxford, MI) according to the manufacturer's protocol to measure MDA and 4-hydroxyalkenals (24), the products upon decomposition of polyunsaturated fatty acid peroxides. Briefly, cells were washed with ice-cold phosphate buffered saline (PBS) once and lysed with 5 mM butylated hydroxytoluene to prevent sample oxidation during preparation. Lysed proteins were incubated with Reagent R1 and 37% HCl for 60 min at 45°C. Proteins were then read at 586 nm wavelength to calculate the concentration based on the standard curve and normalized to the protein concentration measured using separated protein aliquots.

### Protein isolation for oxidized SDS-PAGE and western blot analyses

Before collection, cells were pretreated with or without bosutinib for 1 h, then H_2_O_2_ for 1 h. For whole cell protein lysates, cells were washed twice with ice-cold PBS and were then harvested in M-PER buffer (Pierce, Rockford, IL) with *N*-ethylmaleimide (26), protease inhibitors and phosphatase inhibitors. After quantitation, proteins were run on 4–15% sodium dodecyl sulfate polyacrylamide gel electrophoresis (SDS-PAGE) gels and transferred to nitrocellulose membrane. Blots were then probed with primary antibodies and followed by incubation with secondary antibody and then visualized using enhanced chemiluminescence detection. PTEN antibody was from Cascade Biosciences (6H4.1, Winchester, MA), P-Akt antibody and phospho418-SRC antibody were from Sigma-Aldrich and GAPDH and PARP-1 antibodies were from Santa Cruz Biotech, Santa Cruz, CA).

### Subcellular fractionation

For nuclear-cytoplasmic fractionation, cells were harvested by centrifugation after being trypsinized, and washed with ice-cold PBS twice, and then incubated in buffer A (10 mM HEPES, 1.5 mM KCl, 1% Triton X-100, 1 mM DTT, protein inhibitor cocktail and phosphatase inhibitor) for at least 15 min. Cells were then centrifuged at maximum speed for 20 min at 4°C and supernatant was carefully collected as the cytoplasmic fraction. The pellets were dissolved in ice-cold Nuclear Extraction Reagent (Pierce, Rockford, IL) for 10 min, vortexed on the highest setting for 15 s, returned to ice and continued vortexing for 15 s every 10 min, for a total of 40 min. Centrifuged cells at maximum speed for 15 min, then immediately collected the supernatant (nuclear extract) in new tubes.

### Scratch wound healing assay

FTC133-PTEN wild-type, FTC236-PTEN null and 8505C cells were plated on six-well plates and grew to confluence. The surfaces were scratched with a 10 µl extended length pipette tip and maintained in 37°C for incubation. Each condition was imaged using light microscopy (Leica DMI3000B, Wetzlar, Germany) and analyzed by measuring the distance of the gap over time using Photoshop (Adobe Systems, San Jose, CA).

### Cell cycle analysis by FACS flow cytometry

Cells were serum starved overnight and allowed to grow with or without H_2_O_2_ and with or without bosutinib for 24 s before 70% pre-chilled ethanol fixation and staining with propidium iodide (Roche, Mannheim, Germany). Cell cycle was analyzed with fluorescence-activated cell sorting can flow cytometer (Becton-Dickinson, Franklin Lakes, NJ).

### TUNEL staining and detection

FTC133-PTEN wild-type and FTC236-PTEN null cells were seeded in culture plates with cover slips. After pretreatment with or without bosutinib, cells were exposed to H_2_O_2_ for 1 h. Cells were fixed with freshly prepared 4% paraformaldehyde for 20 min at room temperature, then incubated in freshly prepared permeabilization solution (0.1% Triton X-100, 0.1% sodium citrate) for 2 min on ice. Cells were incubated on cover slips with TUNEL reaction buffer (Roche, Mannheim, Germany) for 1 h at 37°C in a humidified atmosphere in the dark. The cells were subsequently mounted on glass slides with Prolong Gold antifade reagent with DAPI (Invitrogen, Carlsbad, CA) and visualized as previously described (9).

### Statistical analysis

Statistical analysis was performed using Prism for Macintosh (GraphPad) using the two-tailed *t*-test. Data are expressed as medians or means ± SEM as indicated. Statistical significance was identified at *P* < 0.05.

## SUPPLEMENTARY MATERIAL

Supplementary Material is available at *HMG* online.

## FUNDING

This work was supported, in part, by P01CA124570 from the National Cancer Institute, Bethesda, MD (to C.E.) and the Breast Cancer Research Foundation (to C.E.). Y.N. is a CoGEC Scholar funded, in part, by National Cancer Institute grant R25TCA094186. J.N. was an Ambrose Monell Foundation Cancer Genomic Medicine Fellow at the Cleveland Clinic. C.E. is the Sondra J. and Stephen R. Hardis Chair of Cancer Genomic Medicine at the Cleveland Clinic and is an American Cancer Society Clinical Research Professor, generously funded, in part, by the F.M. Kirby Foundation. Funding to pay the Open Access publication charges for this article was provided by the National Cancer Institute.

## Supplementary Material

Supplementary Data
