# Expression of Concern: Cowden syndrome-associated germline *SDHD* variants alter PTEN nuclear translocation through SRC-induced PTEN oxidation

**DOI:** 10.1093/hmg/ddaf042

**Published:** 2025-03-28

This is an expression of concern regarding: Wanfeng Yu, Xin He, Ying Ni, Joanne Ngeow, Charis Eng, Cowden syndrome-associated germline *SDHD* variants alter PTEN nuclear translocation through SRC-induced PTEN oxidation, *Human Molecular Genetics*, Volume 24, Issue 1, 1 January 2015, Pages 142–153, https://doi.org/10.1093/hmg/ddu425.

In September 2024, a reader contacted the journal about similarities in bands in Figure 6A, which were also raised on PubPeer (see https://pubpeer.com/publications/91BB44A9963C29ED0AE3C4F30F4305). The journal has contacted the authors and is investigating these concerns in line with COPE guidance. In the interim, the journal is publishing this Expression of Concern to alert readers while the outcome of the investigation is pending and advises readers to examine the details of this study with particular care.

