# Editor's Note to: Cowden syndrome-associated germline *SDHD* variants alter PTEN nuclear translocation through SRC-induced PTEN oxidation

**DOI:** 10.1093/hmg/ddaf086

**Published:** 2025-05-22

This is an Editor's Note regarding: Wanfeng Yu, Xin He, Ying Ni, Joanne Ngeow, Charis Eng, Cowden syndrome-associated germline *SDHD* variants alter PTEN nuclear translocation through SRC-induced PTEN oxidation, *Human Molecular Genetics*, Volume 24, Issue 1, 1 January 2015, Pages 142–153, https://doi.org/10.1093/hmg/ddu425

In September 2024, a reader contacted the journal about similarities in the P-AKT bands in Figure 6A, which were also raised on PubPeer (see https://pubpeer.com/publications/91BB44A9963C29ED0AE3C4F30F4305). The journal published an expression of concern (1) and investigated the concern in line with COPE guidance.

Wanfeng Yu and Charis Eng are deceased. The remaining co-authors do not have access to the raw data.

Without the original images to review, it is not possible to verify the experimental data presented in Figure 6A. The Editor cautions the reader when interpreting the images presented in Figure 6A and the associated results presented in the article.

The living co-authors were notified of this Editor's Note prior to publication.

**Reference**

1. Expression of Concern: Cowden syndrome-associated germline *SDHD* variants alter PTEN nuclear translocation through SRC-induced PTEN oxidation. *Human Molecular Genetics*, 28 March 2025. ddaf042. https://doi.org/10.1093/hmg/ddaf042

