# Supplementary material for: Cowden syndrome-associated germline SDHD variants alter PTEN nuclear translocation through SRC-induced PTEN oxidation
Source: Hum Mol Genet. 2014 Aug 22;24(1):142–53. doi: 10.1093/hmg/ddu425 (PMC4262496; doi:10.1093/hmg/ddu425)
Supplement: Supplementary Data [file supp_24_1_142__index.html]

Cowden syndrome-associated germline SDHD variants alter PTEN nuclear translocation through SRC-induced PTEN oxidation — Cowden syndrome-associated germline SDHD variants alter PTEN nuclear translocation through SRC-induced PTEN oxidation — Supplementary Data 

# Cowden syndrome-associated germline *SDHD* variants alter PTEN nuclear translocation through SRC-induced PTEN oxidation

## Supplementary Data

Supplementary Data

**Files in this Data Supplement:**

- Supplementary Data - Pdf file
