# Supplementary material for: Cowden syndrome-associated germline SDHD variants alter PTEN nuclear translocation through SRC-induced PTEN oxidation
Source: Hum Mol Genet. 2014 Aug 22;24(1):142–53. doi: 10.1093/hmg/ddu425 (PMC4262496; doi:10.1093/hmg/ddu425)
Supplement: Supplementary Data [file supp_ddu425_ddu425supp.pdf]

SUPPLEMENTAL FIGURE 1

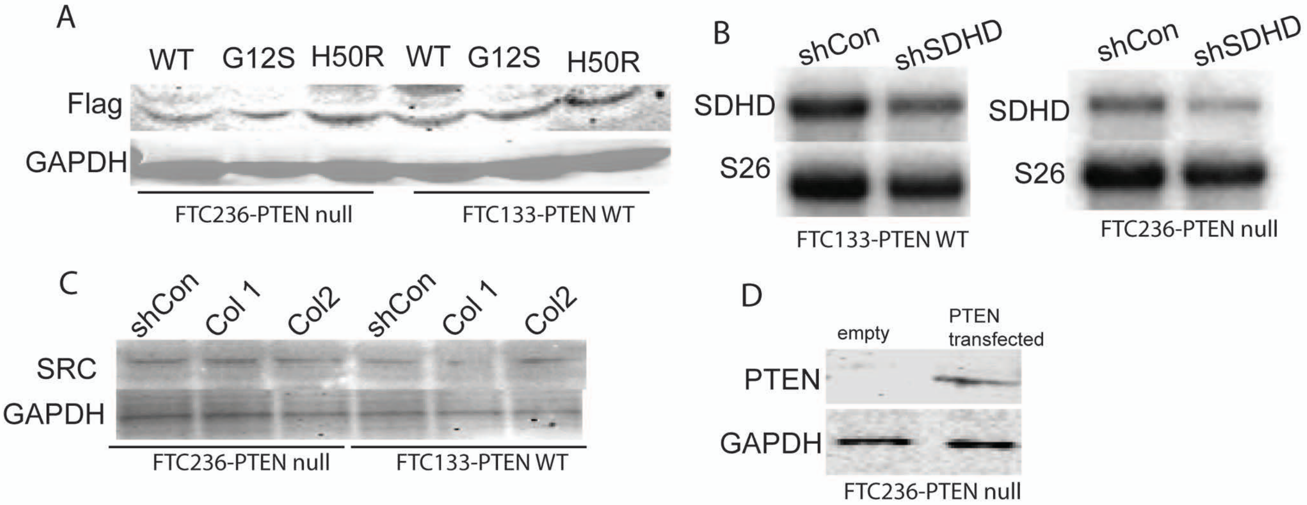

SUPPLEMENTAL FIGURE 2

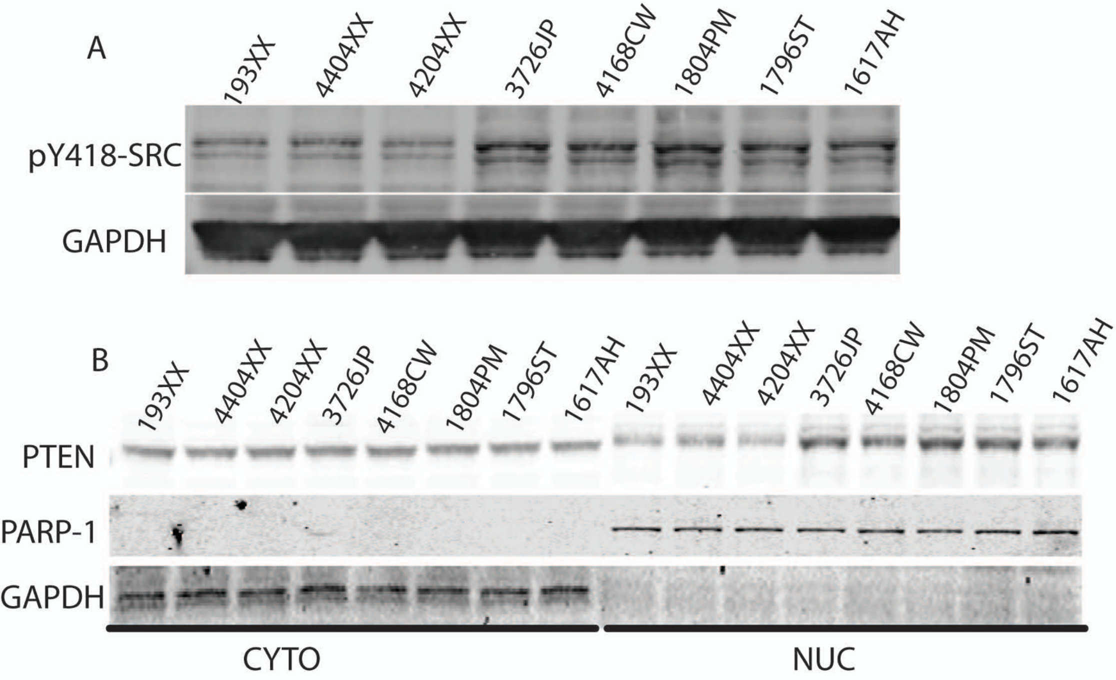

SUPPLEMENTAL LEGENDS TO FIGURES

Supplemental Figure 1. Confirmation of transfection with either Western blot or reverse transcription-PCR. (A) Western blot of Flag-tagged *SDHD*-WT, -G12S and H50R expression in FTC133-PTEN wildtype cells and FTC236-PTEN null cells. (B) Confirmation of PTEN knock down in FTC133-PTEN wildtype and FTC236-PTEN null cells through reverse transcription-PCR. S26 ribosome RNA was used as loading control[30]. (C) Confirmation of SRC knock down in FTC133-PTEN wildtype and FTC236-PTEN null cells with western blot. Colony number.2 (Col 2 in FTC236-PTEN null)) was selected to grow and was used for further experiments. Colony number.1 (Col1 in FTC133-PTEN WT) was selected to grow and used for further experiments. (D) Confirmation of PTEN expression after wildtype PTEN was transfected into FTC236-PTEN null cells.

Supplemental Figure 2. LCL derived from CS patients harboring germline *SDHD* variants (either *SDHD*-G12S or *SDHD*-H50R) showed increased oxidative stress. (A) LCL cells carrying SDHD-G12S or –H50R showed elevated SRC activity compared to control LCL cells. (B) More accumulation of nuclear PTEN was observed in LCL cells carrying SDHD-G12S or –H50R compared to controls. 193XX, 4404XX, 4204XX are LCL controls. LCLs harboring germline *SDHD* H50R: 3726JP, 1804PM, 1796ST; LCLs harboring germline *SDHD* G12S: 4168CW, 1617AH.
